# Ketoacidosis at Hypoglycemic and Low Glucose Levels Associated with SGLT2 Inhibitor Use: A Descriptive Systematic Review of Published Cases

**DOI:** 10.3390/jcm15197595

**Published:** 2026-09-30

**Authors:** Hideya Itagaki, Tomoyuki Endo

**Affiliations:** Division of Emergency and Disaster Medicine, Tohoku Medical and Pharmaceutical University Hospital, 1-12-1 Fukumuro, Miyaginoku, Sendai 983-8512, Miyagi, Japan; entomo@hosp.tohoku-mpu.ac.jp

**Keywords:** SGLT2 inhibitors, ketoacidosis, euglycemic ketoacidosis, hypoglycemia, adverse drug reaction, pharmacovigilance, drug safety, heart failure

## Abstract

**Background/Objectives:** Sodium-glucose cotransporter 2 (SGLT2) inhibitors are increasingly used for heart failure and chronic kidney disease beyond type 2 diabetes, increasing exposure among people without diabetes. Ketoacidosis is a recognized complication, but presentations at low blood glucose (<100 mg/dL) fall outside the familiar euglycemic diabetic ketoacidosis (DKA) alert and are easily missed. We aimed to describe the clinical phenotype of such cases; this review was not registered. **Methods:** We systematically searched PubMed and Web of Science (to 25 June 2026) and included only peer-reviewed case reports and case series describing ketoacidosis with a documented blood glucose <100 mg/dL at or near diagnosis during ongoing SGLT2 inhibitor use or within approximately two weeks after discontinuation. Reporting completeness was assessed with the CARE (Case Report) checklist; analyses were descriptive. **Results:** Fifteen cases met the criteria (median age 61 years; 6/15 nondiabetic; 6/15 receiving it for heart failure). Median glucose was 68 mg/dL (n = 15; 10/15 <70). Metabolic acidosis was frequently marked (median pH 7.17 [n = 14], anion gap 19.9 [n = 13], β-hydroxybutyrate 5.0 mmol/L [n = 11]). Fasting or poor oral intake was the commonest precipitant (11/15). Among six cases with extractable timing, three occurred within 24 h. Blood glucose was not correlated with β-hydroxybutyrate in this small exploratory sample (Spearman ρ = −0.20; n = 11; *p* = 0.56). One death occurred (aspiration). **Conclusions:** This descriptive synthesis characterizes ketoacidosis occurring at hypoglycemic and low glucose levels reported in SGLT2 inhibitor users, including nondiabetic and cardiorenal patients. The design cannot establish incidence, risk, or causality (very low certainty of evidence); clinicians should nonetheless consider ketoacidosis in exposed patients with anion-gap acidosis or ketosis even without hyperglycemia.

## 1. Introduction

Diabetic ketoacidosis (DKA) is a life-threatening endocrine emergency that requires early recognition and treatment [1]. Precipitating factors for diabetic ketoacidosis include new-onset diabetes, inadequate insulin therapy, infection, stroke, myocardial infarction, and pancreatitis; in recent years, sodium-glucose cotransporter 2 (SGLT2) inhibitors have also been recognized as a precipitating factor [2]. SGLT2 inhibitors were initially developed as glucose-lowering agents for type 2 diabetes. However, subsequent large randomized controlled trials demonstrated their prognostic benefits in patients with heart failure and chronic kidney disease, irrespective of diabetes status, and these agents are now widely used to treat heart failure and chronic kidney disease [3,4,5,6]. Accordingly, recent case reports have described clinically significant ketoacidosis during SGLT2 inhibitor therapy not only within the euglycemic range but also at hypoglycemic or near-hypoglycemic glucose levels [1]. Although reports of SGLT2 inhibitor-associated euglycemic DKA have increased, hypoglycemic or low-glucose ketoacidosis occurring at glucose levels below 100 mg/dL has not been sufficiently characterized. Therefore, this study aimed to summarize the clinical characteristics, precipitating factors, diagnoses, treatments, and outcomes of reported cases of hypoglycemic or low-glucose ketoacidosis that occurred during or after SGLT2 inhibitor use. The expanding use of SGLT2 inhibitors for heart failure and chronic kidney disease has increased exposure among people without diabetes, among whom this presentation may be particularly under-recognized. Although several reviews have summarized SGLT2 inhibitor-associated euglycemic diabetic ketoacidosis, to our knowledge, none has specifically characterized presentations at hypoglycemic or low glucose levels (<100 mg/dL); characterizing this least-recognized part of the spectrum is the incremental contribution of the present review. Framed as a PECO question—Population: patients exposed to an SGLT2 inhibitor; Exposure: SGLT2 inhibitor use; Comparator: none; Outcome: ketoacidosis with a documented blood glucose <100 mg/dL at or near diagnosis—this study aimed to describe the clinical and biochemical phenotype of such cases as the primary descriptive outcome, with precipitating factors, a structured causality appraisal, trigger-to-onset intervals, and glucose–ketone and glucose–pH correlations examined as secondary, exploratory analyses.

## 2. Materials and Methods

### 2.1. Protocol and Registration

This systematic review was conducted and reported in accordance with the PRISMA 2020 (Preferred Reporting Items for Systematic Reviews and Meta-Analyses) guidelines [7]. The review was not registered in PROSPERO or any other registry, and no protocol was published or deposited; there is therefore no publicly available evidence of prospective protocol development. The search strategy, eligibility criteria, and outcomes of interest were specified by the authors before data extraction.

### 2.2. Eligibility Criteria

We included peer-reviewed case reports and case series describing patients who developed ketoacidosis during or after exposure to an SGLT2 inhibitor. Eligible reports were required to contain individual patient-level information sufficient to determine the temporal relationship between SGLT2 inhibitor use and the development of ketoacidosis, as well as the clinical course. Full texts were reviewed to extract blood glucose values at or around the time of ketoacidosis diagnosis. No language restriction was applied. We included patients of any age exposed to any SGLT2 inhibitor. Cases were eligible if ketoacidosis occurred during ongoing SGLT2 inhibitor use or within approximately two weeks after discontinuation, reflecting the persistence of the drug’s glucosuric and metabolic effects. For patients with more than one episode, the index episode that met the inclusion criteria was used.

We excluded reviews, editorials, original research articles without relevant case-level data, letters without original case data, clinical trials, observational studies, pharmacoepidemiologic or pharmacovigilance analyses, registry studies, animal or basic science studies, and conference abstracts without sufficient individual patient-level information. Reports in which individual blood glucose values at the time of ketoacidosis diagnosis could not be determined were also excluded.

### 2.3. Information Sources and Search Strategy

A systematic literature search was conducted in PubMed and Web of Science from database inception to 25 June 2026.

PubMed was searched using the following search strategy: ((“Sodium-Glucose Transporter 2 Inhibitors”[Mesh] OR “SGLT2 inhibitor*”[tiab] OR “SGLT-2 inhibitor*”[tiab] OR “sodium-glucose cotransporter 2 inhibitor*”[tiab] OR “sodium glucose cotransporter 2 inhibitor*”[tiab] OR empagliflozin[tiab] OR dapagliflozin[tiab] OR canagliflozin[tiab] OR ertugliflozin[tiab] OR ipragliflozin[tiab] OR tofogliflozin[tiab] OR luseogliflozin[tiab] OR sotagliflozin[tiab]) AND (“Diabetic Ketoacidosis”[Mesh] OR ketoacidosis[tiab] OR keto-acidosis[tiab] OR “diabetic ketoacidosis”[tiab] OR DKA[tiab] OR “euglycemic diabetic ketoacidosis”[tiab] OR “euglycaemic diabetic ketoacidosis”[tiab] OR “euglycemic DKA”[tiab] OR “euglycaemic DKA”[tiab] OR “euglycemic ketoacidosis”[tiab] OR “euglycaemic ketoacidosis”[tiab] OR “normoglycemic ketoacidosis”[tiab]) AND.

(hypoglycemia[tiab] OR hypoglycaemia[tiab] OR hypoglycemic[tiab] OR hypoglycaemic[tiab] OR “low glucose”[tiab] OR “low blood glucose”[tiab] OR “low plasma glucose”[tiab])).

Web of Science was searched using the following search strategy: TS = (SGLT2 OR “SGLT-2” OR empagliflozin OR dapagliflozin OR canagliflozin OR ertugliflozin OR ipragliflozin OR tofogliflozin OR luseogliflozin OR sotagliflozin) AND TS = (ketoacidosis OR “diabetic ketoacidosis” OR DKA OR “euglycemic ketoacidosis” OR “euglycaemic ketoacidosis” OR “euglycemic DKA” OR “euglycaemic DKA”)

AND TS = (“case report” OR “case series” OR “case presentation” OR “we report”).

Records from both databases were screened by title and abstract to identify potentially eligible studies, and the full texts of potentially relevant articles were assessed for eligibility. Because blood glucose values are rarely reported in titles, abstracts, or indexing terms, the low-glucose criterion (<100 mg/dL at or near ketoacidosis diagnosis) was applied uniformly at the full-text eligibility stage rather than embedded in the database queries; retrieval of all previously known sentinel cases was confirmed. No language restriction was applied. In addition, backward citation searching (screening the reference lists of the included reports and relevant reviews) was performed to identify further eligible studies; forward citation searching was not performed. PubMed and Web of Science were selected because together they index the general medical, emergency, and subspecialty journals in which such case reports are published, and they capture all previously known sentinel cases; the restriction to the two databases and the absence of Embase, Scopus, or grey-literature sources are acknowledged as limitations.

### 2.4. Study Selection

Records were managed in EndNote, and duplicate records were removed using its duplicate-detection function with manual confirmation. Following a screening pilot to calibrate the criteria, two reviewers (H.I. and T.E.) independently screened titles and abstracts, and the full texts were then assessed by the same two reviewers. Studies not meeting the inclusion/exclusion criteria were excluded, and any disagreements were resolved through discussion and consensus. No additional eligible records were identified through reference-list (backward citation) screening.

### 2.5. Data Extraction and Data Items

Data were extracted from eligible reports using a standardized data extraction form. Extracted variables included author, publication year, patient age and sex, diabetes status or diabetes type, indication for SGLT2 inhibitor use, type of SGLT2 inhibitor, duration of treatment, precipitating factors or clinical context, blood glucose level at or around the time of ketoacidosis diagnosis, pH, serum bicarbonate level, anion gap, ketone measurements, treatment, clinical course, and outcome. Patient-level data were extracted by one reviewer (H.I.) and independently verified against the source articles by a second reviewer (T.E.), with discrepancies resolved by consensus.

When multiple glucose values were reported, the value measured at or closest to the time of ketoacidosis diagnosis was used. If the timing of glucose measurement was unclear, it was recorded, and cases in which the blood glucose level at the time of ketoacidosis diagnosis could not be determined were excluded from the main analysis.

### 2.6. Causality Assessment

Causality was descriptively appraised using the World Health Organization–Uppsala Monitoring Centre (WHO–UMC) system by considering the temporal relationship between SGLT2 inhibitor exposure and ketoacidosis onset, the presence of alternative or competing explanations, and the response to SGLT2 inhibitor discontinuation when reported. The assessment was intended to describe the plausibility of a contributory relationship and was not used to establish definitive causality; the per-case appraisal is provided in Table S4.

### 2.7. Reporting Completeness Assessment

Reporting completeness of the included case reports and case series was assessed using the CARE (CAse REport) checklist [8], a reporting guideline rather than a risk-of-bias instrument. Each of the 30 CARE items was scored as fully reported (○ = 1), partially reported (△ = 0.5), or not reported/unclear (x = 0), and the item scores were summed. The assessment was performed by one reviewer and independently verified by a second, with disagreements resolved by consensus. CARE scores are reported descriptively as an indicator of reporting completeness; because CARE does not define validated quality thresholds, reports are not classified as high or low methodological quality.

### 2.8. Certainty of Evidence Assessment

The overall certainty of the body of evidence was appraised using a Grading of Recommendations Assessment, Development, and Evaluation (GRADE)-informed approach [9]. Because the evidence base comprised uncontrolled individual case reports and case series, the starting certainty was rated as very low, consistent with GRADE guidance for non-comparative observational data. We further considered limitations, including risk of publication and selective-reporting bias, indirectness arising from heterogeneous patient populations and indications, and imprecision due to the small number of cases and incomplete biochemical data. Given the descriptive, non-comparative nature of the synthesis, formal Summary of Findings tables were not constructed; the certainty of the overall body of evidence was instead summarized narratively.

### 2.9. Definitions and Diagnostic Criteria

Ketoacidosis was defined based on the diagnoses and biochemical findings reported in the original articles, including metabolic acidosis, low bicarbonate level, increased anion gap, and positive blood or urine ketones. Blood glucose values were extracted at or around the time of ketoacidosis diagnosis. For inclusion, ketoacidosis had to be diagnosed in the source article and supported by metabolic acidosis (low serum bicarbonate and/or low blood pH with an increased anion gap) together with documented ketosis (positive blood or urine ketones and/or elevated β-hydroxybutyrate), in addition to a documented blood glucose <100 mg/dL at or near diagnosis; all 15 included cases met this definition. Throughout, “diabetic ketoacidosis (DKA)” is used only for patients with established diabetes and “ketoacidosis” otherwise. To operationalize the diagnosis prospectively, in addition to a ketoacidosis diagnosis in the source article, we required objective evidence of (i) metabolic acidosis (a reduced blood pH and/or a reduced serum bicarbonate relative to the reporting laboratory’s reference range); (ii) an increased anion gap where an anion gap was reported; and (iii) ketosis (an elevated blood β-hydroxybutyrate and/or at least moderate ketonuria). Because pH and bicarbonate were reported from arterial, venous, or unspecified samples and against differing reference ranges, we did not impose a single fixed pH or bicarbonate cut-off; instead, we required documented acidosis together with objective ketosis and, where available, an increased anion gap. Across the 15 included cases, the reported blood pH ranged from <6.8 to 7.34, serum bicarbonate from below the detectable limit to 19.1 mmol/L, the anion gap (when reported) from 13.8 to 35 mmol/L, and β-hydroxybutyrate from 1.7 to 9.1 mmol/L. When acidosis could be attributed in part to a competing process—lactic acidosis, advanced hepatic disease, starvation ketosis, or alcoholic ketoacidosis—the case was retained only if ketoacidosis during SGLT2 inhibitor exposure was documented and the acidosis and ketosis criteria above were met; such coexisting processes were recorded as competing explanations rather than used as grounds for exclusion. For cases with mixed acid–base disturbances, eligibility was based on the presence of a ketoacidotic component meeting the criteria above; the two cases with markedly elevated lactate (17 and 19.7 mmol/L) also had documented ketosis and anion-gap acidosis and were retained on that basis.

These glucose-based categories are investigator-defined operational classifications used for descriptive purposes in this review and are not established diagnostic entities. For descriptive classification, ketoacidosis with hypoglycemia was defined as a blood glucose level <70 mg/dL and ketoacidosis with low glucose as 70–99 mg/dL; these labels denote ketoacidosis coinciding with the specified glucose range rather than a distinct diagnosis. For comparison, glucose levels of 100–249 mg/dL were categorized as the euglycemic range—consistent with commonly used euglycemic-DKA thresholds (glucose < 200–250 mg/dL)—and levels ≥250 mg/dL as hyperglycemic diabetic ketoacidosis. The main analysis focused on cases with glucose <100 mg/dL. Conceptually, these categories extend the established notion of euglycemic DKA toward the lower end of the glucose spectrum rather than defining a separate disease.

### 2.10. Statistical Analysis

Descriptive statistics were calculated using Microsoft Excel. Continuous variables were summarized as medians with interquartile ranges. Values reported with inequality signs were handled conservatively by using the reported boundary value for descriptive analyses (for example, <6.8 was entered as 6.8 and >8.0 as 8.0); values described only as below the detectable limit or not reported were treated as missing. Associations between continuous variables were assessed with Spearman’s rank correlation coefficient; 95% confidence intervals were derived using the Fisher z-transformation, and exact two-sided *p*-values are reported. All analyses were exploratory and descriptive; no formal hypothesis testing was performed, and no significance thresholds were applied.

## 3. Results

**Figure 1:**
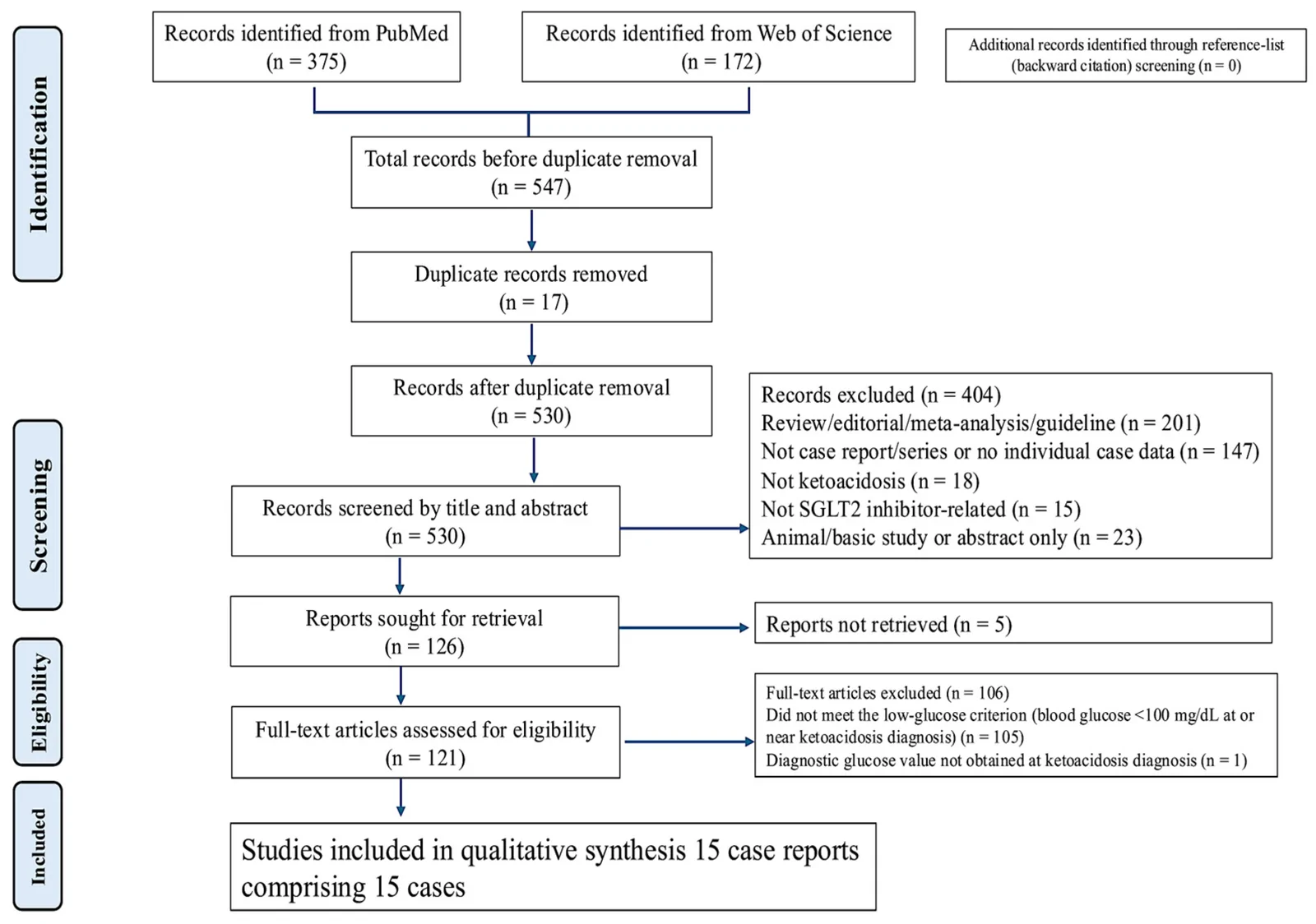
Preferred Reporting Items for Systematic Reviews and Meta-Analyses (PRISMA) 2020 flow diagram of study identification, screening, and inclusion.

A total of 547 records were identified (375 from PubMed and 172 from Web of Science; Figure 1). After removing 17 duplicates, 530 records were screened by title and abstract, and 404 were excluded. The full texts of 126 reports were sought, of which 5 could not be retrieved, leaving 121 full-text articles assessed for eligibility. Of these, 106 were excluded: 105 because blood glucose was ≥100 mg/dL at or near ketoacidosis diagnosis, i.e., they did not meet the low-glucose criterion (<100 mg/dL), and 1 (Meier 2025 [10]) because no case in that series had a documented blood glucose <100 mg/dL at ketoacidosis diagnosis (Case 1, 239 mg/dL; Case 3, 346 mg/dL at admission and 155 mg/dL at recurrence; Case 2, 94 mg/dL but with pH 7.36 and bicarbonate 19.8 mmol/L, i.e., not meeting the ketoacidosis definition at that low-glucose time point). Fifteen case reports comprising 15 cases were included in the qualitative synthesis [1,11,12,13,14,15,16,17,18,19,20,21,22,23,24].

### 3.1. Case Characteristics and Biochemical Features

The characteristics of the 15 main-analysis cases are summarized in Table 1, with detailed case-level information provided in Table S1.

Ten cases had ketoacidosis with hypoglycemia (glucose < 70 mg/dL) and five had ketoacidosis with low glucose (70–99 mg/dL). The median age was 61 years (interquartile range [IQR], 36–71), and 6 patients (40.0%) were female. Type 2 diabetes was present in 8 cases (53.3%), latent autoimmune diabetes in adults (LADA) in 1 case (6.7%), and no diabetes or a nondiabetic status in 6 cases (40.0%). The indication for SGLT2 inhibitor therapy was diabetes-related in 9 cases (60.0%) and heart failure (congestive heart failure) in 6 cases (40.0%). Empagliflozin was the most frequently reported SGLT2 inhibitor, used in 11 cases (73.3%), followed by dapagliflozin in 3 cases (20.0%) and canagliflozin in 1 case (6.7%).

Glucose levels ranged from 11 to 98 mg/dL, with a median of 68.0 mg/dL (IQR, 54.0–79.5). Ten cases (66.7%) had ketoacidosis with hypoglycemia (glucose < 70 mg/dL), and 5 cases (33.3%) had ketoacidosis with low glucose (70–99 mg/dL).

Despite the absence of hyperglycemia, clinically meaningful metabolic acidosis was observed, with a median pH of 7.17 (IQR, 7.13–7.27), a median bicarbonate level of 12.1 mmol/L (IQR, 11.0–15.5), and a median anion gap of 19.9 (IQR, 17.0–23.7).

Among cases with available β-hydroxybutyrate measurements, substantial ketonemia was observed, with values ranging from 1.7 to 9.1 mmol/L and a median value of 5.0 mmol/L. Urine ketones were positive in all evaluable cases in which they were reported, although reporting formats varied across reports. Lactate levels were available in 11 cases, with a median of 1.32 mmol/L (range, 0.5–19.7 mmol/L). Although marked hyperlactatemia was reported in two cases, most evaluable cases had normal or only mildly elevated lactate levels.

In an exploratory analysis, blood glucose at diagnosis was not correlated with β-hydroxybutyrate among cases with paired data (Spearman ρ = −0.20; 95% confidence interval (CI), −0.71 to 0.45; *p* = 0.56; n = 11; Table S3). A positive correlation was observed between blood glucose and reported blood pH (Spearman ρ = 0.58; 95% CI, 0.07 to 0.85; *p* = 0.03; n = 14; Table S3). Given the small number of evaluable cases and incomplete paired data, these analyses are exploratory and descriptive and are not interpreted inferentially.

Fasting or poor oral intake was the most frequently reported precipitating factor or clinical context, occurring in 11 cases (73.3%). Other contexts included heart failure background in 6 cases (40.0%), surgery or perioperative states in 3 cases (20.0%), low-carbohydrate or ketogenic diets and alcohol-related contexts in 2 of 13 evaluable cases each (15.4%), infection or acute illness in 2 cases (13.3%), and pancreatitis in 1 case (6.7%).

An interval from the precipitating event to ketoacidosis could be extracted or reasonably inferred in only 6 of the 15 cases; among these, ketoacidosis developed within 24 h in 3, within >24 h to ≤2 days in 1, and within >2 to ≤7 days in 2. In the remaining cases, the interval could not be determined. Because these intervals were few and partly inferred, they are reported descriptively and do not permit conclusions about the tempo of onset.

Regarding management and outcomes, dextrose administration was reported in all evaluable cases, reflecting the need to treat ketoacidosis despite low or low–normal glucose levels. Insulin was used in 10 of 14 evaluable cases (71.4%), and SGLT2 inhibitors were discontinued in all 13 evaluable cases. intensive care unit (ICU) admission was reported in 8 cases (53.3%). Invasive interventions were uncommon: intubation and continuous renal replacement therapy (CRRT) or dialysis were each required in 1 of 14 evaluable cases. One death was reported.

### 3.2. Diagnostic Clues and Competing Clinical Explanations

Diagnostic clue patterns and competing clinical explanations are summarized in Table 2. Elevated ketones in blood or urine were the most frequent diagnostic clue pattern, identified in 13 cases. Additional clue patterns included glucosuria despite low or normal glucose levels in 4 cases and a persistent anion gap despite treatment of an alternative suspected cause in 3 cases.

Competing diagnoses or alternative clinical explanations were common and were not mutually exclusive. The most frequent was starvation- or diet-related ketosis, identified in 7 cases, followed by hepatic disease, glycogen storage disease, or low-glycogen states in 4 cases, and dehydration, acute kidney injury, or shock in 3 cases. Other competing explanations included alcoholic ketoacidosis, cardiac events or procedures, biliary or pancreatic disease, infection or acute illness, surgical or perioperative contexts, drug-induced hypoglycemia, and insulin withdrawal or relative insulin deficiency.

In the descriptive WHO–UMC appraisal, 11 of the 15 cases were categorized as probable and 4 as possible (Table S4). The four possible cases comprised two with alcohol involvement, in which alcoholic ketoacidosis was a plausible independent explanation, and two in which the response to SGLT2 inhibitor discontinuation was not clearly reported. No case met the criteria for a certain categorization, as rechallenge was not performed in any report. This appraisal was descriptive and was not used to establish definitive causality.

### 3.3. Reporting Completeness

Reporting completeness, assessed with the CARE checklist (Table S2), was generally high: the median CARE score was 25 (IQR, 24–26; range 21–28). Using descriptive cut-offs (defined only for presentation and not as quality thresholds), 10 reports had a score ≥25 and 5 had a score of 18–24. Commonly underreported items included patient perspective, formal timelines, follow-up details, and explicit informed consent statements.

### 3.4. Certainty of Evidence

Using a GRADE-informed approach, the overall certainty of the body of evidence was rated very low. This reflects the uncontrolled case-report and case-series study design, the potential for publication and selective-reporting bias, indirectness across heterogeneous populations and indications, and imprecision arising from the small number of cases and incomplete paired biochemical data. Accordingly, the findings should be interpreted as hypothesis-generating rather than as establishing causal or quantitative relationships.

## 4. Discussion

In this descriptive systematic review of published cases, we characterized the clinical phenotype of SGLT2 inhibitor-associated ketoacidosis occurring at hypoglycemic or low glucose levels. Across 15 reported cases, clinically meaningful ketoacidosis was present despite blood glucose below 100 mg/dL, with substantial ketonemia in cases with available β-hydroxybutyrate measurements. These cases were not confined to the classic at-risk profile: Notably, 6 of 15 patients were nondiabetic, and 6 of 15 were receiving an SGLT2 inhibitor for heart failure, consistent with the fact that the expanding use of SGLT2 inhibitors for heart failure and chronic kidney disease has increased exposure among people without diabetes. Fasting or poor oral intake was the most frequently reported precipitating factor. Because a dissociation between glycemia and ketoacidosis severity—well recognized at near-normal (“euglycemic”) glucose—was also observed with frank hypoglycemia (glucose <70 mg/dL in 10 of 15 cases), clinicians should not be reassured by low glucose values when ketoacidosis is otherwise suspected. These observations are descriptive and, given the design, cannot establish incidence, relative risk, or causality.

These case-level observations are consistent with a recognized class-level association between SGLT2 inhibitors and ketoacidosis reported in pharmacovigilance databases. Disproportionality analyses have described signals for SGLT2 inhibitor-associated ketoacidosis in the WHO VigiBase, the US Food and Drug Administration (FDA) Adverse Event Reporting System, and the Japanese Adverse Drug Event Report (JADER) database, the last yielding a reporting odds ratio of 72.2 (95% CI 59.3–87.8) [25,26,27]. However, spontaneous-reporting databases generally do not capture laboratory values such as blood glucose in a structured or complete manner and therefore cannot reliably distinguish hyperglycemic from euglycemic or hypoglycemic presentations. Characterizing the low-glucose phenotype described here therefore relies on case-level synthesis, although such synthesis cannot itself quantify frequency or risk.

A possible pathophysiological framework is that, similar to mechanisms proposed for SGLT2 inhibitor-associated euglycemic ketoacidosis, exposure to SGLT2 inhibitors may promote a starvation-like metabolic state under conditions of reduced carbohydrate availability. SGLT2 inhibitors promote urinary glucose excretion and lower circulating glucose levels, which may reduce glucose-dependent endogenous insulin secretion and, through an increased glucagon-to-insulin ratio, promote lipolysis and hepatic ketone production [28,29]. When this state is accompanied by fasting, poor oral intake, low carbohydrate intake, alcohol exposure, perioperative stress, heart failure, or acute illness, hepatic glycogen depletion, increased lipolysis, and relative insulin deficiency may be further amplified, potentially increasing ketone production. This mechanism may explain why clinically significant ketoacidosis can develop even when blood glucose levels are low or in the low–normal range.

In an exploratory analysis, blood glucose was not correlated with β-hydroxybutyrate (Spearman ρ = −0.20; 95% CI, −0.71 to 0.45; *p* = 0.56; n = 11), indicating that, in this small sample, the degree of ketonemia could not be predicted from blood glucose alone [30]; this observation is hypothesis-generating rather than confirmatory. A weak-to-moderate positive association was observed between blood glucose and reported blood pH (Spearman ρ = 0.58; 95% CI, 0.07 to 0.85; *p* = 0.03; n = 14). This association is exploratory, based on few cases with incomplete and heterogeneously sourced (arterial, venous, or unspecified) pH data, and should not be interpreted as evidence that lower glucose causes more severe acidemia. Overall, these findings are consistent with assessing ketones and acid–base status separately in SGLT2 inhibitor-exposed patients even in the absence of hyperglycemia.

Data on the interval from the precipitating event to onset were available in only 6 of 15 cases; among these, three developed ketoacidosis within 24 h. Given the small number of evaluable and partly inferred intervals and the absence of a comparator group, these data are insufficient to characterize the tempo of onset, and we report them descriptively without inferring a characteristically rapid course. Nonetheless, clinicians should remain alert to ketoacidosis from the onset of fasting, surgery, infection, or other acute stressors in exposed patients.

These reports illustrate how difficult this condition can be to recognize. Competing explanations—starvation ketosis, alcohol-related ketosis, liver disease, dehydration, shock, infection, and perioperative stress—often coexist and can mask SGLT2 inhibitor involvement. Two clues may be useful: glucosuria despite hypoglycemia or normoglycemia suggests ongoing SGLT2 inhibitor action, and a persistent anion-gap acidosis after administration of glucose and treatment of alternative causes should prompt measurement of blood ketones and reassessment for SGLT2 inhibitor-associated ketoacidosis.

Established management and preventive guidance are also relevant. Acute ketoacidosis should be managed according to contemporary diabetic ketoacidosis and euglycemic-DKA guidance [31]. In addition, regulatory labeling recommends withholding SGLT2 inhibitors before scheduled surgery (canagliflozin, dapagliflozin, and empagliflozin 3 days, and ertugliflozin at least 4 days, before the procedure) [32], and current multidisciplinary and international guidance advises temporary discontinuation during acute illness, prolonged fasting, or the perioperative period, together with a low threshold for ketone testing [33]. Although this guidance is directed largely at euglycemic and hyperglycemic presentations, our observations reinforce that the same precautions and diagnostic vigilance apply when glucose is low; this review does not itself generate treatment recommendations.

Regarding management, dextrose was reported in all evaluable cases and insulin in 10 of 14. Although the cases managed without insulin tended to have higher pH values and did not require ICU admission, whereas insulin was used in more severe cases, this pattern reflects confounding by indication—more severely affected patients received insulin because of their severity—and these reports cannot determine which patients can be safely managed without insulin. We therefore refrain from inferring treatment thresholds from this series; the review describes reported management patterns rather than comparative effectiveness, and treatment should follow contemporary DKA and euglycemic-DKA guidance, individualized to acid–base status, ketonemia, hemodynamic condition, and glucose concentration—generally including discontinuation of the SGLT2 inhibitor, correction of dehydration and precipitating factors, carbohydrate/dextrose administration, insulin when indicated, and monitoring of acid–base status and ketones [32,33].

This review has several limitations. The analysis was based on published case reports, and the findings are subject to publication bias, incomplete reporting, and heterogeneity in diagnostic evaluation. The number of included cases was small, and exploratory correlation analyses were limited by missing paired biochemical data. Trigger-to-ketoacidosis intervals could be clearly extracted or reasonably inferred in only a minority of cases. In addition, β-hydroxybutyrate, lactate, urine ketones, and treatment details were not reported uniformly across studies. Therefore, the proposed pathophysiological interpretation should be considered hypothesis-generating rather than definitive. In formal terms, the overall certainty of the body of evidence, appraised using a GRADE-informed approach, was very low, reflecting the uncontrolled case-report design, risk of publication and reporting bias, indirectness, and imprecision. Additional limitations include the restriction to two databases (PubMed and Web of Science) and to published, predominantly English-language reports, as a result of which the study may have missed unpublished or non-indexed cases and is subject to selective reporting of biochemical data. The timing of glucose measurement relative to diagnosis and to treatment (including dextrose administration) was not uniformly reported, which may affect the classification of glucose levels. Because the included reports are uncontrolled and often feature coexisting fasting, alcohol use, or hepatic disease, drug-associated ketoacidosis cannot be reliably distinguished from starvation- or alcohol-related ketoacidosis. Finally, the absence of a denominator precludes any estimate of frequency, incidence, or comparative risk.

## 5. Conclusions

In conclusion, this descriptive synthesis characterizes ketoacidosis occurring at hypoglycemic and low glucose levels reported in patients using SGLT2 inhibitors, in which ketone-driven metabolic acidosis can occur despite low or low–normal blood glucose. Such cases have been reported in nondiabetic and cardiorenal users who fall outside the traditional ketoacidosis-risk profile; however, because the evidence comprises a small number of published case reports (very low certainty), their frequency, relative risk, and predictive factors cannot be estimated, and this review does not establish a causal association or quantify the magnitude of risk. Clinicians should nonetheless consider ketoacidosis in any SGLT2 inhibitor-exposed patient with anion-gap metabolic acidosis, ketosis, or glucosuria even in the absence of hyperglycemia, and prospective, denominator-based studies are needed to establish incidence, risk factors, and outcomes.

## Figures and Tables

**Table 1:** Baseline characteristics and ketoacidosis-related features of SGLT2 inhibitor-associated ketoacidosis at hypoglycemic and low glucose levels.

Parameters	Overall (n = 15)
**Clinical characteristics**	
Age, median (IQR)	61 (36–71)
Female sex, n (%)	6 (40.0%)
**Diabetes status**	
Type 2 DM, n (%)	8 (53.3%)
LADA, n (%)	1 (6.7%)
No diabetes/nondiabetic, n (%)	6 (40.0%)
**Indication for SGLT2 inhibitor**	
Diabetes-related indication, n (%)	9 (60%)
Heart failure, n (%)	6 (40%)
**SGLT2 inhibitor**	
Empagliflozin, n (%)	11 (73.3%)
Dapagliflozin, n (%)	3 (20.0%)
Canagliflozin, n (%)	1 (6.7%)
**Glucose category**	
Glucose range, mg/dL	11–98
Glucose, median (IQR), mg/dL	68.0 (54.0–79.5)
Hypoglycemic ketoacidosis, glucose < 70 mg/dL, n (%)	10 (66.7%)
Low-glucose ketoacidosis, glucose 70–99 mg/dL, n (%)	5 (33.3%)
**Ketoacidosis severity**	
pH, median (IQR)	7.17 (7.13–7.27)
Bicarbonate, median (IQR), mmol/L	12.1 (11.0–15.5)
Anion gap, median (IQR)	19.9 (17.0–23.7)
β-Hydroxybutyrate available, n (%)	11/15 (73.3%)
β-Hydroxybutyrate, median (IQR), mmol/L	5.0 (4.2–8.1)
β-Hydroxybutyrate range, mmol/L	1.7–9.1
Urine ketone positive, n/evaluable (%)	11/11 (100%)
Lactate available, n (%)	11/15 (73.3%)
Lactate, median (IQR), mmol/L	1.32 (0.95–2.9)
Lactate range, mmol/L	0.5–19.7
**Precipitating factors/clinical contexts**	
Fasting or poor oral intake, n (%)	11 (73.3%)
Low carbohydrate/ketogenic diet, n (%)	2/13 (15.4%)
Alcohol-related context, n (%)	2/13 (15.4%)
Surgery or perioperative state, n (%)	3 (20.0%)
Infection/acute illness, n (%)	2 (13.3%)
Pancreatitis, n (%)	1 (6.7%)
Heart failure background, n (%)	6 (40.0%)
**Trigger-to-ketoacidosis interval**	
Interval clearly reported or reasonably inferable	6 (40.0%)
≤24 h	3 (20.0%)
24 h to ≤2 days	1 (6.7%)
>2 to ≤7 days	2 (13.3%)
>7 days	0 (0.0%)
Unclear/not extractable	9 (60.0%)
**Management and outcomes**	
Dextrose used, n (%)	14/14 (100%)
Insulin used, n (%)	10/14 (71.4%)
SGLT2 inhibitor discontinued, n (%)	13/13 (100%)
ICU admission, n (%)	8/15 (53.3%)
Intubation, n (%)	1/14 (7.1%)
CRRT or dialysis, n (%)	1/14 (7.1%)
Death, n (%)	1/15 (6.7%)

Notes: Data are presented as No. (%) unless otherwise indicated. Percentages were calculated using the number of evaluable cases for each variable. Ketoacidosis with hypoglycemia was defined as a glucose level <70 mg/dL, and ketoacidosis with low glucose as 70–99 mg/dL at or near the time of ketoacidosis diagnosis; these are investigator-defined descriptive categories rather than distinct diagnoses. β-Hydroxybutyrate and lactate values were summarized among cases with available measurements. Urine ketone results were reported qualitatively or semi-quantitatively and were summarized as positive among evaluable cases. Precipitating factors and clinical contexts were categorized based on descriptions in the original case reports and were not mutually exclusive. Trigger-to-ketoacidosis interval was categorized only when the interval from a reported trigger to ketoacidosis diagnosis was clearly reported or reasonably inferable. For pH, one value reported as <6.8 was treated as 6.8 for descriptive calculation. pH values are presented as reported in the original articles and may include arterial, venous, or unspecified blood gas measurements. Abbreviations: CRRT, continuous renal replacement therapy; DKA, diabetic ketoacidosis; IQR, interquartile range; LADA, latent autoimmune diabetes in adults; SGLT2, sodium–glucose cotransporter 2.

**Table 2:** Diagnostic clues and competing clinical explanations in hypoglycemic and low-glucose SGLT2 inhibitor-associated ketoacidosis.

Findings	Hypoglycemic n = 10	Low-Glucose n = 5	Total n = 15
**Diagnostic clue patterns**			
Ketone elevation (blood/urine)	8	5	13
Glucosuria despite low/normal glucose	2	2	4
Persistent anion gap despite treatment of alternative cause	3	0	3
**Competing diagnoses/alternative clinical explanations**			
Starvation/diet-related ketosis	5	2	7
Hepatic disease/glycogen storage or low-glycogen state	3	1	4
Dehydration/AKI/shock	2	1	3
Alcoholic ketoacidosis	2	0	2
Cardiac event/procedure	1	1	2
Biliary/pancreatic disease	2	0	2
Infection/acute illness	1	1	2
Surgical or perioperative context	1	1	2
Drug-induced hypoglycemia	1	0	1
Insulin withdrawal/relative insulin deficiency	0	1	1

Notes: Data are presented as No. Categories were based on diagnostic narratives in the original case reports and were not mutually exclusive. Diagnostic clue patterns refer to findings that prompted or supported recognition of ketoacidosis despite low or low–normal glucose levels. Competing diagnoses or alternative clinical explanations refer to alternative diagnoses, clinical contexts, or precipitating conditions that could have obscured recognition of SGLT2 inhibitor-associated ketoacidosis. Abbreviations: AKI, acute kidney injury; SGLT2, sodium–glucose cotransporter 2.

## Data Availability

All data analyzed in this systematic review were extracted from previously published, publicly available case reports cited herein; the extracted dataset is provided in the Supplementary Materials.

## Supplementary Materials

The following supporting information can be downloaded at https://www.mdpi.com/article/10.3390/jcm15197595/s1, File S1: PRISMA 2020 checklist; File S2: Full reproducible search strategies; Table S1: Case-level data; Table S2: CARE reporting-completeness assessment; Table S3: Exploratory Correlation Analysis Between Blood Glucose and Biochemical Severity Markers; Table S4: Case-level causality appraisal (WHO–UMC system).

## Abbreviations

CARE	CAse REport (guidelines)
CRRT	Continuous renal replacement therapy
DKA	Diabetic ketoacidosis
ICU	Intensive care unit
IQR	Interquartile range
LADA	Latent autoimmune diabetes in adults
PRISMA	Preferred Reporting Items for Systematic Reviews and Meta-Analyses
SGLT2	Sodium–glucose cotransporter 2
