# Supplementary material for: Ketoacidosis at Hypoglycemic and Low Glucose Levels Associated with SGLT2 Inhibitor Use: A Descriptive Systematic Review of Published Cases
Source: J Clin Med. 2026 Sep 30;15(19):7595. doi: 10.3390/jcm15197595 (PMC13665874; doi:10.3390/jcm15197595)
Supplement: Supplementary file 1 [file jcm-15-07595-s001.zip › File S1 PRISMA 2020 checklist.pdf]

PRISMA 2020 Checklist

Manuscript: "Ketoacidosis without hyperglycemia as an under-recognized safety signal of SGLT2 inhibitors: a systematic review of published cases"

Note: "Location" gives the manuscript section where each item is reported; insert final page/line numbers before submission. From: Page MJ, et al. The PRISMA 2020 statement. BMJ 2021;372:n71.

Section/Topic	#	Checklist item	Location
**TITLE**			
Title	1	Identify the report as a systematic review.	Title page
**ABSTRACT**			
Abstract	2	See the PRISMA 2020 for Abstracts checklist.	Abstract
**INTRODUCTION**			
Rationale	3	Describe the rationale for the review in the context of existing knowledge.	Introduction (paras 1–2)
Objectives	4	Provide an explicit statement of the objective(s) or question(s) the review addresses.	Introduction (final para)
**METHODS**			
Eligibility criteria	5	Specify the inclusion and exclusion criteria and how studies were grouped for the syntheses.	Methods – Eligibility criteria; Definitions and Diagnostic Criteria
Information sources	6	Specify all databases/sources searched and the date each was last searched.	Methods – Information sources and search strategy (PubMed, Web of Science; to 25 Jun 2026)
Search strategy	7	Present the full search strategies for all databases, including any filters/limits.	Methods – Information sources and search strategy (full PubMed and WoS strings)
Selection process	8	Specify methods to decide if a study met criteria, how many reviewers, and if independent.	Methods – Study Selection (two reviewers, independent, consensus)
Data collection process	9	Specify methods to collect data from reports, how many reviewers, and if independent.	Methods – Data Extraction and Data Items (standardized form; two reviewers)
Data items	10a	List and define all outcomes for which data were sought.	Methods – Data Extraction and Data Items (glucose, pH, HCO3, anion gap, ketones, treatment, outcome)
Data items	10b	List and define all other variables for which data were sought.	Methods – Data Extraction and Data Items (age, sex, diabetes status, indication, agent, duration, precipitants)
Study risk of bias assessment	11	Specify methods used to assess risk of bias / reporting quality in included studies.	Methods – Quality Assessment (CARE checklist; two reviewers, independent, consensus)
Effect measures	12	Specify the effect measure(s) used for each outcome.	Not applicable – descriptive synthesis (medians/IQR, correlation); no pooled effect measures. Methods – Definitions
Synthesis methods	13a	Describe processes to decide which studies were eligible for each synthesis.	Methods – Definitions and Diagnostic Criteria (glucose-based groupings; main analysis <100 mg/dL)
Synthesis methods	13b	Describe methods to prepare data for presentation/synthesis.	Methods – Data Extraction and Data Items (value nearest to diagnosis used)
Synthesis methods	13c	Describe methods to tabulate or visually display results.	Results – Tables 1–2; Supplementary Tables 1–3
Synthesis methods	13d	Describe methods used to synthesize results and the rationale.	Narrative synthesis (medians/IQR, exploratory correlations); no meta-analysis due to heterogeneity/small n. Methods; Discussion (limitations)
Synthesis methods	13e	Describe methods to explore causes of heterogeneity.	Exploratory correlation analysis only. Results – correlation; Supplementary Table 3

Synthesis methods	13f	Describe any sensitivity analyses.	Not applicable (no formal sensitivity analysis; one case excluded from main analysis, Results)
Reporting bias assessment	14	Describe methods to assess risk of bias due to missing results in a synthesis.	Not formally assessed; publication/reporting bias addressed narratively. Discussion – limitations
Certainty assessment	15	Describe methods to assess certainty (confidence) in the body of evidence.	Methods – Certainty of Evidence (GRADE-informed; starting certainty very low for uncontrolled case-report evidence)
**RESULTS**			
Study selection	16a	Describe the results of the search and selection, ideally with a flow diagram.	Results (para 1); Figure 1 (PRISMA flow diagram)
Study selection	16b	Cite studies that seemed to meet criteria but were excluded, and explain why.	Results (exclusion counts/reasons); one case excluded (Meier 2025, Case 2); Figure 1
Study characteristics	17	Cite each included study and present its characteristics.	Table 1; Supplementary Table 1
Risk of bias in studies	18	Present assessments of risk of bias / reporting quality for each included study.	Results – Reporting completeness; Supplementary Table 2 (CARE)
Results of individual studies	19	Present summary statistics for each study for all outcomes.	Supplementary Table 1 (case-level data)
Results of syntheses	20a	Summarise characteristics and risk of bias among contributing studies.	Results – Case characteristics; Reporting completeness
Results of syntheses	20b	Present results of all statistical syntheses conducted.	Results – biochemical features (medians/IQR); correlation results
Results of syntheses	20c	Present results of investigations of possible causes of heterogeneity.	Results – exploratory correlations; Supplementary Table 3
Results of syntheses	20d	Present results of all sensitivity analyses.	Not applicable
Reporting biases	21	Present assessments of risk of bias due to missing results.	Discussion – limitations (publication/reporting bias)
Certainty of evidence	22	Present assessments of certainty in the body of evidence.	Results – Certainty of evidence (overall very low); Discussion – limitations
**DISCUSSION**			
Discussion	23a	Provide a general interpretation of the results in the context of other evidence.	Discussion (paras 1–4)
Discussion	23b	Discuss limitations of the evidence included in the review.	Discussion – limitations
Discussion	23c	Discuss limitations of the review processes used.	Discussion – limitations (no pre-registration, small n, missing data)
Discussion	23d	Discuss implications for practice, policy, and future research.	Discussion (final paras); Conclusions
**OTHER INFORMATION**			
Registration and protocol	24a	Provide registration information, or state the review was not registered.	Methods – Protocol and Registration (not registered in PROSPERO)
Registration and protocol	24b	Indicate where the protocol can be accessed, or state none was prepared.	Methods – Protocol and Registration (a priori methods; no separate protocol)
Registration and protocol	24c	Describe and explain any amendments to registration/protocol.	Not applicable (none)
Support	25	Describe sources of financial/non-financial support and the role of funders.	Declarations – Funding (no funding received)
Competing interests	26	Declare any competing interests of review authors.	Declarations – Competing interests (none)
Availability of data, code and other materials	27	Report which materials are publicly available and where.	Data availability statement; Supplementary Tables 1–3 (extracted data); data from published sources
