# Supplementary material for: Ketoacidosis at Hypoglycemic and Low Glucose Levels Associated with SGLT2 Inhibitor Use: A Descriptive Systematic Review of Published Cases
Source: J Clin Med. 2026 Sep 30;15(19):7595. doi: 10.3390/jcm15197595 (PMC13665874; doi:10.3390/jcm15197595)
Supplement: Supplementary file 1 [file jcm-15-07595-s001.zip › File S2 Full reproducible search strategies.pdf]

File S2. Full reproducible search strategies

This file provides the complete, reproducible search strategies for the systematic review, together with database sources, field tags, filters, language settings, deduplication method, and results per source.

Databases and final search date

Sources: PubMed (NLM) and Web of Science Core Collection. Coverage: database inception to the final search date. Final search date: 25 June 2026. No language restriction. No date or publication-type filters were applied within the database queries (case-report/case-series restriction was applied within the Web of Science string and, for PubMed, at the full-text eligibility stage). Records per source before deduplication: PubMed = 375; Web of Science = 172 (total 547). Duplicates removed = 17; records after deduplication = 530.

Deduplication

Records from both databases were combined in EndNote; duplicates were removed using the EndNote duplicate-detection function followed by manual verification.

Low-glucose eligibility criterion

Because blood glucose values are rarely reported in titles, abstracts, or indexing terms, the low-glucose criterion (blood glucose <100 mg/dL at or near ketoacidosis diagnosis) was applied uniformly at the full-text eligibility stage rather than embedded in the database queries. Retrieval of all previously known sentinel cases was confirmed.

PubMed search strategy (field tags shown)

```
(("Sodium-Glucose Transporter 2 Inhibitors"[Mesh] OR "SGLT2 inhibitor*"[tiab] OR "SGLT-2 inhibitor*"[tiab] OR "sodium-glucose cotransporter 2 inhibitor*"[tiab] OR "sodium glucose cotransporter 2 inhibitor*"[tiab] OR empagliflozin[tiab] OR dapagliflozin[tiab] OR canagliflozin[tiab] OR ertugliflozin[tiab] OR ipragliflozin[tiab] OR tofogliflozin[tiab] OR luseogliflozin[tiab] OR sotagliflozin[tiab]) AND ("Diabetic Ketoacidosis"[Mesh] OR ketoacidosis[tiab] OR keto-acidosis[tiab] OR "diabetic ketoacidosis"[tiab] OR DKA[tiab] OR "euglycemic diabetic ketoacidosis"[tiab] OR "euglycaemic diabetic ketoacidosis"[tiab] OR "euglycemic DKA"[tiab] OR "euglycaemic DKA"[tiab] OR "euglycemic ketoacidosis"[tiab] OR "euglycaemic ketoacidosis"[tiab] OR "normoglycemic ketoacidosis"[tiab]) AND (hypoglycemia[tiab] OR hypoglycaemia[tiab] OR hypoglycemic[tiab] OR hypoglycaemic[tiab] OR "low glucose"[tiab] OR "low blood glucose"[tiab] OR "low plasma glucose"[tiab]))
```

Web of Science (Core Collection) search strategy (TS = Topic)

```
TS=(SGLT2 OR "SGLT-2" OR empagliflozin OR dapagliflozin OR canagliflozin OR ertugliflozin OR ipragliflozin OR tofogliflozin OR luseogliflozin OR sotagliflozin) AND TS=(ketoacidosis OR "diabetic ketoacidosis" OR DKA OR "euglycemic ketoacidosis" OR "euglycaemic ketoacidosis" OR "euglycemic DKA" OR "euglycaemic DKA") AND TS=("case report" OR "case series" OR "case presentation" OR "we report")
```

Supplementary searching

Backward citation searching (screening the reference lists of included reports and relevant reviews) was performed; no additional eligible records were identified. Forward citation searching was not performed.

Justification for two databases

PubMed and Web of Science together index the general medical, emergency, and subspecialty journals in which the relevant case reports are published and captured all previously known sentinel cases. The restriction to two databases and the absence of Embase, Scopus, and grey-literature sources are acknowledged as limitations.
