# Supplementary material for: Ketoacidosis at Hypoglycemic and Low Glucose Levels Associated with SGLT2 Inhibitor Use: A Descriptive Systematic Review of Published Cases
Source: J Clin Med. 2026 Sep 30;15(19):7595. doi: 10.3390/jcm15197595 (PMC13665874; doi:10.3390/jcm15197595)
Supplement: Supplementary file 1 [file jcm-15-07595-s001.zip › Table S3.pdf]

Supplementary Table S3. Exploratory Correlation Analysis Between Blood Glucose and Biochemical Severity Markers

Variable pair	No. of cases	Spearman ρ	95% CI	p-value	Interpretation
Blood glucose vs β-hydroxybutyrate	11	−0.20	−0.71 to 0.45	0.56	No correlation
Blood glucose vs blood pH	14	0.58	0.07 to 0.85	0.03	Weak-to-moderate positive correlation

Notes: Associations were assessed using the Spearman rank correlation coefficient among cases with available paired quantitative data; cases with missing or non-quantitative values were excluded from each analysis. Ninety-five percent confidence intervals were derived using the Fisher z-transformation, and exact two-sided p-values are reported. Blood pH values were reported as arterial, venous, or unspecified in the original articles. Because of the small number of evaluable cases and incomplete paired data, these analyses are exploratory and hypothesis-generating and are not interpreted inferentially.
