# Supplementary material for: Ketoacidosis at Hypoglycemic and Low Glucose Levels Associated with SGLT2 Inhibitor Use: A Descriptive Systematic Review of Published Cases
Source: J Clin Med. 2026 Sep 30;15(19):7595. doi: 10.3390/jcm15197595 (PMC13665874; doi:10.3390/jcm15197595)
Supplement: Supplementary file 1 [file jcm-15-07595-s001.zip › Table S4.pdf]

Table S4. Case-level causality appraisal (WHO–UMC system)

WHO–UMC categories reflect a descriptive causality appraisal based on the temporal relationship, competing/alternative explanations, and dechallenge information reported in each source. Alcohol-related cases (Iesaka 2023; Dhoop 2025) were categorized as Possible because alcoholic ketoacidosis is a plausible independent explanation. Cases without a clearly reported dechallenge response (Kang 2022; Johnson 2026) were also categorized as Possible, as a reasonable dechallenge could not be confirmed. All other cases had a reasonable temporal relationship, precipitants rather than sufficient alternative diagnoses, and a reported clinical recovery after discontinuation, and were categorized as Probable. This appraisal is descriptive and was not used to establish definitive causality.

First author (Year)	Temporal relationship	Alternative/competing explanations	Dechallenge (discontinuation and response)	WHO–UMC category
Kelmenson 2017	Ketoacidosis during ongoing SGLT2 inhibitor use, following fasting/poor intake	fasting/poor intake	SGLT2 inhibitor discontinued; clinical recovery reported	Probable
O'Neill 2020	Ketoacidosis during ongoing SGLT2 inhibitor use, following infection	infection	SGLT2 inhibitor discontinued; clinical recovery reported	Probable
Dorcely 2021	Ketoacidosis during ongoing SGLT2 inhibitor use, following fasting/poor intake, low-carbohydrate diet	fasting/poor intake; low-carbohydrate diet	SGLT2 inhibitor discontinued; clinical recovery reported	Probable
Iesaka 2023	Ketoacidosis during ongoing SGLT2 inhibitor use, following alcohol, heart failure	alcohol	SGLT2 inhibitor discontinued; clinical recovery reported	Possible
Seki 2022	Ketoacidosis during ongoing SGLT2 inhibitor use, following fasting/poor intake, surgery/perioperative, heart failure	fasting/poor intake; surgery/perioperative	SGLT2 inhibitor discontinued; clinical recovery reported	Probable
Kang 2022	Ketoacidosis during ongoing SGLT2 inhibitor use, following fasting/poor intake, low-carbohydrate diet, surgery/perioperative	fasting/poor intake; low-carbohydrate diet; surgery/perioperative	Not clearly reported	Possible (dechallenge not clearly reported)
Tomita 2025	Ketoacidosis during ongoing SGLT2 inhibitor use, following fasting/poor intake, heart failure	fasting/poor intake	SGLT2 inhibitor discontinued; clinical recovery reported	Probable
Yoshida 2025	Ketoacidosis during ongoing SGLT2 inhibitor use, following fasting/poor intake	fasting/poor intake	SGLT2 inhibitor discontinued; clinical recovery reported	Probable
Alsulaimi 2026	Ketoacidosis during ongoing SGLT2 inhibitor use, following fasting/poor intake, surgery/perioperative	fasting/poor intake; surgery/perioperative	SGLT2 inhibitor discontinued; clinical recovery reported	Probable
Dhoop 2025	Ketoacidosis during ongoing SGLT2 inhibitor use, following alcohol, heart failure	alcohol; hepatic/alcohol-related	SGLT2 inhibitor discontinued; clinical recovery reported	Possible
Tito 2024	Ketoacidosis during ongoing SGLT2 inhibitor use, following fasting/poor intake	fasting/poor intake	SGLT2 inhibitor discontinued; clinical recovery reported	Probable
Sydney 2025	Ketoacidosis during ongoing SGLT2 inhibitor use, following pancreatitis	pancreatitis	SGLT2 inhibitor discontinued; clinical recovery reported	Probable
Gutiérrez-Baena 2025	Ketoacidosis during ongoing SGLT2 inhibitor use, following fasting/poor intake, infection	fasting/poor intake; infection	SGLT2 inhibitor discontinued; clinical recovery reported	Probable
Trapanese 2024	Ketoacidosis during ongoing SGLT2 inhibitor use, following fasting/poor intake, heart failure	fasting/poor intake	SGLT2 inhibitor discontinued; clinical recovery reported	Probable
Johnson 2026	Ketoacidosis during ongoing SGLT2 inhibitor use, following fasting/poor intake, infection, heart failure	fasting/poor intake; infection	Not clearly reported	Possible (dechallenge not clearly reported)
